# Case Report: Dupilumab-associated ulcerative colitis: elucidating the pathomechanistic link between Th2 blockade and Th17 polarized intestinal inflammation

**DOI:** 10.3389/fimmu.2026.1875748

**Published:** 2026-07-08

**Authors:** Fanghui Fu, Qing Zhao, Lei Ma, Aili Wang, Zhe Wang, Xiaoyang Xu

**Affiliations:** 1 Department of Dermatology, Binzhou Medical University Hospital, Binzhou, Shandong, China; 2 Department of Dermatology, Hospital for Skin Diseases, Shandong First Medical University, Jinan, Shandong, China; 3 Department of Gastroenterology, Binzhou Medical University Hospital, Binzhou, Shandong, China; 4 Department of Dermatology, Shandong Provincial Hospital Affiliated to Shandong First Medical University, Jinan, Shandong, China; 5 Department of Pathology, Binzhou Medical University Hospital, Binzhou, Shandong, China

**Keywords:** atopic dermatitis, dupilumab, immune shift, Th2/Th17 cytokines, ulcerative colitis

## Abstract

While dupilumab is highly effective in managing moderate-to-severe atopic dermatitis (AD) through targeted IL-4/IL-13 receptor antagonism, its broader immunomodulatory effects warrant careful clinical scrutiny. We report the case of a 63-year-old male who developed ulcerative colitis (UC) following dupilumab therapy. Although his cutaneous condition improved rapidly, the patient developed acute gastrointestinal distress, including hematochezia and tenesmus, within three months of initiating therapy. Subsequent colonoscopy and histopathological analyses confirmed the diagnosis of UC. Discontinuation of dupilumab was followed by a robust clinical and endoscopic remission. Longitudinal immunohistochemical profiling of the colonic mucosa demonstrated elevated IL-17 and suppressed IL-4 expression during active colitis, which normalized upon clinical recovery. These findings offer hypothesis-generating evidence indicative of a localized Th2-to-Th17 immune shift. Although inherently limited as a single-patient report, this case underscores the critical necessity for multidisciplinary vigilance regarding paradoxical Th17-driven inflammation in patients undergoing IL-4Rα blockade.

## Introduction

Atopic dermatitis (AD) is a prevalent, chronic, relapsing inflammatory skin disease primarily driven by type 2 (Th2) immune responses. The immunological signature of AD is characterized by profound barrier dysfunction coupled with an exaggerated expression of Th2 cytokines, notably interleukin-4 (IL-4) and interleukin-13 (IL-13) (1). The introduction of dupilumab, a monoclonal antibody that selectively inhibits the IL-4Rα subunit, has provided a potent mechanism to dampen these hyperactive inflammatory cascades. By obstructing the shared receptor component for IL-4 and IL-13, dupilumab effectively neutralizes the downstream signaling pathways that sustain cutaneous inflammation, making it a primary therapeutic option for moderate-to-severe AD that remains refractory to conventional modalities (2). However, the precision of such potent immunological interventions can invariably disrupt broader homeostatic networks. Emerging evidence links its use to the onset of Th17-mediated diseases within the spondyloarthropathy spectrum (SpA), such as psoriasis and uveitis, leading clinical immunologists to posit an immune shift effect. This hypothesis suggests that Th2 cytokines typically function as a physiological brake on Th17-mediated inflammation, maintaining a delicate equilibrium that can be abruptly destabilized by therapeutic interventions targeting a single pathway (3, 4).

While rheumatological manifestations are documented, the specific impact of IL-4Rα antagonism on the gastrointestinal mucosal immune system remains incompletely characterized (5). Although ulcerative colitis (UC) is defined by chronic Th17-associated inflammation, a direct causal relationship between dupilumab and UC has yet to be rigorously substantiated (6). Current literature only hints at a theoretical risk based on sporadic case reports (7, 8), and lacks granular histological validation correlating systemic drug exposure with local intestinal cytokine perturbations at the tissue level.

In this report, we describe a case of dupilumab-associated UC in which clinical remission was directly linked to drug cessation. Through localized immunohistochemical mapping, we offer mechanistic insights into how systemic Th2 blockade may precipitate severe, localized Th17-driven intestinal inflammation.

## Case presentation

**Figure 1:**
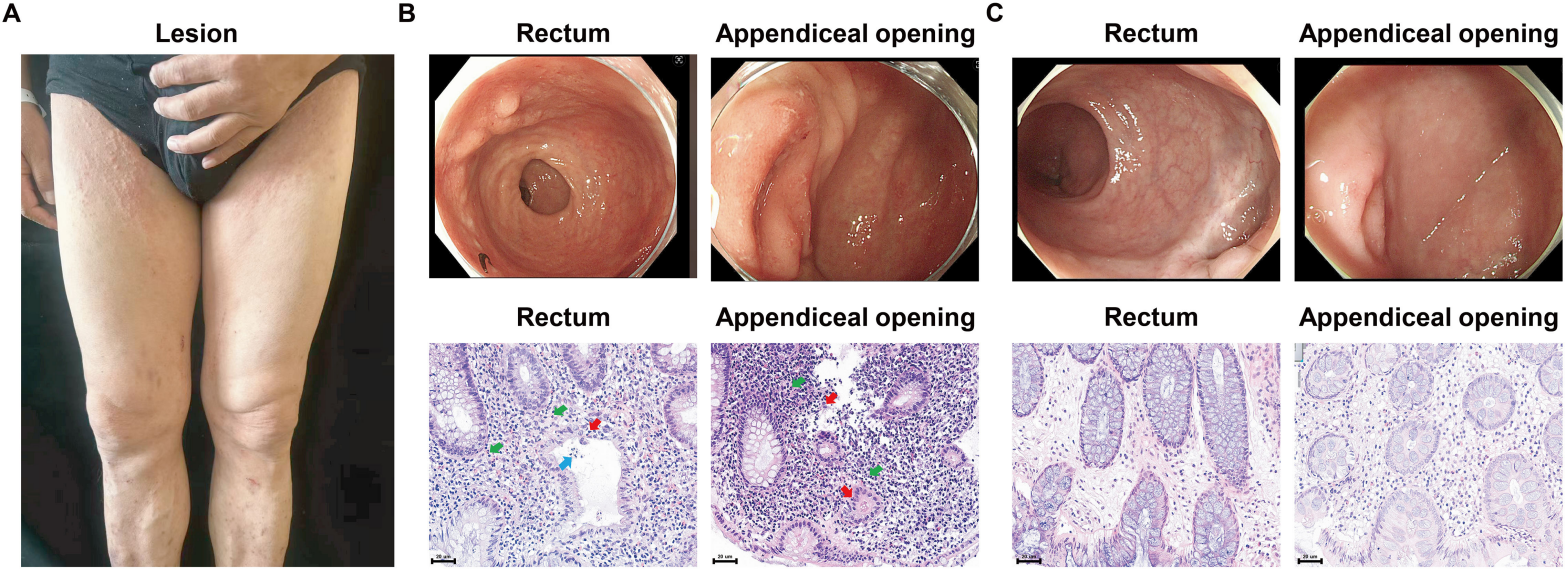
Endoscopic image and histological features of patient. **(A)** The typical skin lesions of the patient. **(B)** Endoscopic examination and HE staining (bar = 0.02 mm) revealed obvious inflammation at both rectum and appendiceal opening after dupilumab administration. Green arrows indicate abundant infiltration of neutrophils; red arrows indicate the destruction of glandular structures; and the blue arrow indicates a sinus abscess. **(C)** Endoscopic examination and HE staining showed that the inflammation had significantly decreased at both rectum and appendiceal opening after 5 months of dupilumab discontinuation.

A 63-year-old man with a 6-year history of refractory, widespread erythematous-papular eruptions was referred to our hospital. Upon presentation, physical examination revealed widespread erythema, papules, and excoriations with marked popliteal lichenification (**Figure 1A**), corresponding to a baseline Eczema Area and Severity Index (EASI) of 13.3. He was diagnosed with moderate-to-severe AD and given his complete lack of response to conventional therapies, including topical corticosteroids and systemic antihistamines, targeted biological therapy was initiated.

Dupilumab was initially administered at a loading dose of 600 mg, followed by a maintenance dose of 300 mg biweekly, while concurrently discontinuing all prior pharmacotherapies except for basic emollients. The cutaneous response was marked; the patient achieved rapid dermatological clearance at 4 weeks (EASI: 7) and near-complete remission by 12 weeks (EASI: 1). However, during the 12th week of treatment, the patient developed acute and severe gastrointestinal manifestations characterized by abdominal pain, tenesmus, and hematochezia. A comprehensive temporal and medical history effectively excluded alternative triggers: he reported no familial history of inflammatory bowel disease (IBD), no tobacco or alcohol consumption, and no recent exposure to NSAIDs or novel oral medications. Furthermore, a screening colonoscopy performed 5 years prior was entirely unremarkable. An urgent colonoscopy revealed loss of normal vascular patterns, superficial granularity, and mucosal erosions extending from the rectal mucosa to the appendiceal orifice, corresponding to a Mayo Endoscopic Subscore (MES) of 1. Histopathological analysis of the aforementioned sites demonstrated robust localized inflammation, characterized by mixed inflammatory cell infiltrates, active cryptitis, and architectural distortion (**Figure 1B**).

To rigorously exclude infectious or ischemic etiologies, an exhaustive diagnostic workup was performed. Stool cultures, ova and parasite exams, cytomegalovirus (CMV) DNA quantification, and *Clostridioides difficile* toxin assays were uniformly negative. Systemic inflammatory markers (WBC, CRP, ESR) remained surprisingly within normal limits without evidence of anemia; however, fecal calprotectin was markedly elevated at 200 µg/g, serving as an objective biochemical indicator of active, localized intestinal inflammation.

These clinicopathological parameters supported a definitive diagnosis of UC. Formal causality assessment utilizing the Naranjo adverse drug reaction probability scale yielded a “probable” association (**Table 1**). Given this temporal association, dupilumab therapy was immediately discontinued. Notably, this targeted dechallenge resulted in a complete resolution of all gastrointestinal symptoms without the necessity of introducing systemic corticosteroids, mesalamine, or other conventional IBD pharmacotherapies. A follow-up colonoscopy evaluating the previously affected segments, performed 5 months post-cessation, confirmed robust endoscopic and histological healing of the colonic mucosa (**Figure 1C**).

**Table 1:** Naranjo adverse drug reaction probability scale.

Question	Yes	No	Do not know	Score
Are there previous conclusive reports on this reaction?	+1	0	0	1
Did the adverse event appear after the suspected drug was administered?	+2	-1	0	2
Did the adverse reaction improve when the drug was discontinued or a specific antagonist was administered?	+1	0	0	1
Did the adverse event reappear when the drug was re-administered?	+2	-1	0	0
Are there alternative causes (other than the drug) that could on their own have caused the reaction?	-1	+2	0	2
Did the reaction reappear when a placebo was given?	-1	+1	0	0
Was the drug detected in blood (or other fluids) in concentrations known to be toxic?	+1	0	0	0
Was the reaction more severe when the dose was increased or less severe when the dose was decreased?	+1	0	0	0
Did the patient have a similar reaction to the same or similar drugs in any previous exposure?	+1	0	0	0
Was the adverse event confirmed by any objective evidence?	+1	0	0	1
**Total score**				7

Scoring: Definite: >9; Probable: 5–8: Possible: 1–4; Doubtful: 0.

## Immunohistochemical examination

**Figure 2:**
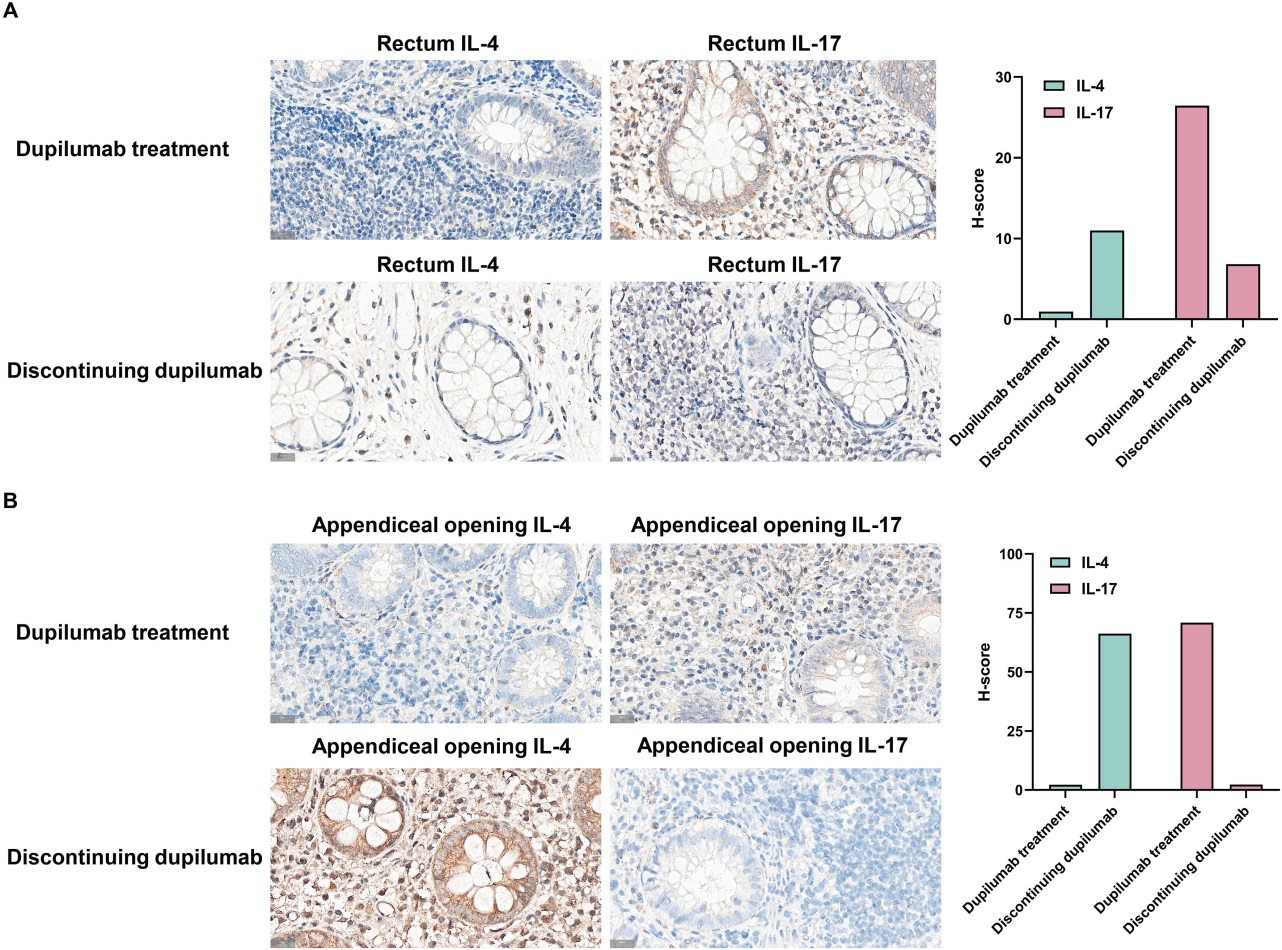
Expression patterns of IL-4 and IL-17 before and after dupilumab discontinuation. **(A)** Immunohistochemical analysis illustrating the expression patterns of IL-4 and IL-17 in rectum obtained from patients before and after dupilumab discontinuation (bar = 0.02 mm). **(B)** Immunohistochemical analysis illustrating the expression patterns of IL-4 and IL-17 in appendiceal opening before and after dupilumab discontinuation (bar = 0.02 mm).

To probe the potential functional implications of the Th2 and Th17 pathways of patients with UC, immunohistochemical analysis of the appendix and rectum was performed. This detailed histological profiling showed elevated IL-17 and suppressed IL-4 expression during the active stage of the colitis (**Figures 2A, B**). Strikingly, these patterns completely reversed at the recovery stage: IL-4 was significantly upregulated, while IL-17 was downregulated (**Figures 2A, B**). This immunohistochemical reversal correlated with the observed clinical remission of symptoms in the patient subsequent to the withdrawal of dupilumab treatment.

## Discussion

The clinical trajectory of this case highlights the intricate networks of immunological cross-talk potentially unmasked by targeted therapy. Although previous literature has reported sporadic cases of ulcerative colitis temporally associated with dupilumab therapy, the present case adds to these reports by providing a longitudinal, tissue-level evaluation of IL-4 and IL-17 expression patterns during active colitis and subsequent clinical remission. Our findings offer supporting evidence suggesting that IL-4Rα blockade may be temporally associated with the disinhibition of IL-17 production, a mechanism congruent with reported Th17-mediated dermatological and rheumatic events (9).

UC is characterized by chronic intestinal inflammation driven, in part, by Th17 cells and IL-17 (10). The inverse relationship between Th2 and Th17 cytokines in the intestinal mucosa supports the concept that IL-4Rα blockade may disinhibit IL-17-driven inflammation (11, 12). This phenomenon is rooted in the immune shift hypothesis, which posits that the Th17 and Th2 cellular pathways are mutually antagonistic at both the transcriptional and functional levels. Dominance of one immune axis typically leads to the suppression of the other (13). Under physiological conditions, Th2 cytokines act as a regulatory brake on Th17 polarization. Consequently, therapeutic inhibition of the Th2 axis by dupilumab may inadvertently remove this homeostatic suppression of the Th17 axis.

Our histopathological findings provide localized evidence of this potential systemic shift. We observed an apparent inverse correlation between IL-17 and IL-4 expression within the colonic mucosa, which correlated clinically with the patient’s disease course. Despite these compelling histological observations, several limitations must be acknowledged. First, due to the lack of baseline IL-17 and IL-4 levels prior to dupilumab initiation, it is difficult to assess definitively whether subclinical immune dysregulation predated the treatment. Consequently, whether the Th2 blockade unmasked a pre-existing mucosal imbalance or induced the inflammation *de novo* remains to be elucidated. Second, the observed alterations in IL-17 and IL-4 expression might partially reflect non-specific shifts in the general inflammatory burden rather than a strictly dupilumab-driven pathomechanism. To establish definitive cell lineage dynamics and confirm causal pathway relationships, further high-resolution analyses, such as multiplex immunofluorescence, flow cytometry, transcriptomics, or single-cell RNA sequencing, are warranted.

Although gastrointestinal adverse events are not typically highlighted in dupilumab’s primary safety profile, this case mandates increased vigilance (14). Clinicians should maintain a high index of suspicion for Th17-driven inflammation in patients presenting with new-onset chronic diarrhea or hematochezia during IL-4/IL-13 inhibitor therapy. Early recognition and multidisciplinary assessment involving both dermatologists and gastroenterologists remain the cornerstone of management. Although cessation of the offending biologic was sufficient to induce remission in this specific patient, clinicians should be aware that this approach may not be universally effective, and individualized supportive UC therapies may be required.

In conclusion, we report a clinically relevant case of dupilumab-associated UC, supported by immunohistochemistry suggesting a localized Th2-to-Th17 shift. Future large-scale cohort studies are required to quantify this risk and delineate predictive biomarkers that could identify at-risk patients prior to the initiation of therapy.

## Data Availability

The original contributions presented in the study are included in the article/supplementary material. Further inquiries can be directed to the corresponding authors.
